# Sensitive and Rapid Dual One‐Tube Recombinase Aided PCR Assays for the Simultaneous Detection of Cytomegalovirus, Epstein‐Barr Virus, Human Herpesvirus 6, and Herpes Simplex Virus 1/2 in Whole Blood From Children Undergoing Hematopoietic Stem Cell Transplantation

**DOI:** 10.1002/jmv.70905

**Published:** 2026-04-07

**Authors:** Li Zhao, Mengchuan Zhao, Yuan Gao, Yanqing Tie, Yinghui Guo, Shuai Zhang, Yuxin Wang, Deran Ma, Zhili Shao, Zhishan Feng

**Affiliations:** ^1^ Department of Clinical Laboratory Diagnosis Hebei Medical University Shijiazhuang Hebei China; ^2^ Department of Blood Transfusion Children's Hospital of Hebei Province Shijiazhuang Hebei China; ^3^ Hebei Provincial Clinical Research Center for Child Health and Disease Shijiazhuang Hebei China; ^4^ Department of Clinical Laboratory Hebei General Hospital Shijiazhuang Hebei China; ^5^ Hebei Key Laboratory of Molecular Medicine Shijiazhuang Hebei China; ^6^ Hebei Clinical Research Center for Laboratory Medicine Shijiazhuang Hebei China; ^7^ Department of Clinical Laboratory Children's Hospital of Hebei Province Shijiazhuang Hebei China; ^8^ Department of Clinical Laboratory Shijiazhuang People's Hospital Shijiazhuang Hebei China

**Keywords:** hematopoietic stem cell transplantation, herpesviruses, pediatric patients, recombinase‐aided PCR

## Abstract

Early, rapid, and sensitive detection of cytomegalovirus (CMV), Epstein‐Barr virus (EBV), human herpesvirus 6 (HHV‐6), and herpes simplex virus Types 1/2 (HSV‐1/2) is critical for improving clinical outcomes in pediatric hematopoietic stem cell transplantation (HSCT). A dual one‐tube recombinase‐aided PCR (DO‐RAP) assay was developed for the simultaneous detection of these viruses. The analytical sensitivity and specificity of the assay were evaluated and subsequently validated using 70 clinical whole blood samples. The DO‐RAP assay demonstrated limits of detection (LOD) of two copies/reaction for CMV and HHV‐6, 7 copies/reaction for EBV, and three copies/reaction for HSV‐1/2. The total detection time was 62 min. In whole blood samples, DO‐RAP demonstrated 100% sensitivity relative to qPCR, and identified 37 additional positive cases among qPCR‐negative samples. DO‐RAP exhibited higher sensitivity than qPCR for monitoring viral status before HSCT, at 0–30 days post‐HSCT, and at 31–60 days post‐HSCT, enabling earlier detection of CMV, EBV, HHV‐6, and HSV‐1/2 in recipients. During donor screening, DO‐RAP also identified one additional CMV‐positive and EBV‐positive case compared with qPCR. The DO‐RAP assay enables rapid and accurate detection of low‐level viral infections in whole blood samples from HSCT settings, providing a valuable tool for routine monitoring of HSCT‐associated viral infections.

## 1 Introduction

Hematopoietic stem cell transplantation (HSCT) is an effective treatment for a variety of hematological malignancies and non‐malignant diseases; however, viral infections remain a major cause of morbidity and mortality after transplantation [1, 2]. During HSCT, factors such as donor source, severe neutropenia, and immunosuppressive therapy significantly increase the risk of viral infection and reactivation in recipients [3]. Among these pathogens, herpesviruses represent the most common opportunistic infections in HSCT recipients, including cytomegalovirus (CMV), Epstein‐Barr virus (EBV), human herpesvirus 6 (HHV‐6), and herpes simplex virus types 1/2 (HSV‐1/2). CMV infects 45.5%–85% of healthy individuals and can cause severe pneumonia and enteritis after transplantation, with mortality rates exceeding 50% [4, 5, 6]. EBV infection may lead to post‐transplantation lymphoproliferative disorder (PTLD) [7]. HHV‐6 infection is associated with high mortality, and presents with a wide spectrum of clinical manifestations, including fever, rash, bone marrow suppression, hepatitis, pneumonitis, and encephalitis [8]. HSV infections account for nearly 50% of oropharyngeal lesions during the early post‐HSCT period [9]. Timely detection and monitoring of these viruses are therefore essential for improving transplant outcomes and patient survival.

In recent years, metagenomic next‐generation sequencing (mNGS) [10] has gained increasing use in clinical practice due to its ability to detect unknown pathogens; however, its reliance on highly trained personnel and its substantial cost limit widespread implementation in routine clinical laboratories. While traditional real‐time quantitative PCR (qPCR) [11] and multiplex qPCR [12, 13] are routinely used for viral infection detection, their sensitivity remains inadequate for clinical samples with low viral loads. Recombinase‐aided amplification (RAA) is a rapid and highly sensitive isothermal amplification technique; however, its broader application is constrained by the complexity of probe design and limited throughput capacity.

In this study, RAA was integrated with qPCR to develop a novel dual one‐tube recombinase‐aided PCR (DO‐RAP) assay for the detection of four viruses, offering enhanced sensitivity and a shorter turn‐around time (TAT). This method facilitates early identification, prevention, and management of viral infections, which is essential for reducing both the incidence of infection and mortality following HSCT, thereby improving transplantation success and patient survival.

## 2 Materials and Methods

### 2.1 Clinical Samples and Nucleic Acid Extraction

**Figure 1:**
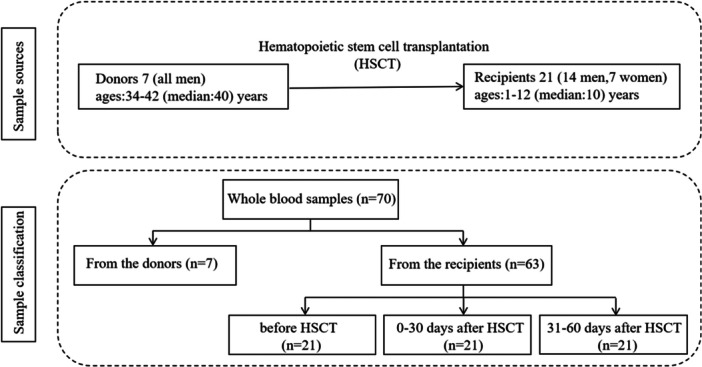
Flowchart of sample information.

A total of 7 HSCT donors and 21 HSCT recipients were enrolled at the Children's Hospital of Hebei Province (China) between April 2024 and April 2025. The study included 70 EDTA whole blood samples collected from donors, recipients prior to HSCT, recipients within 30 days post‐HSCT, and recipients within 31–60 days after HSCT (Figure 1). Briefly, 1.8 mL of EDTA whole blood samples were collected into 2 mL of transport medium and stored at −80°C. Among the HSCT recipients, 10 were diagnosed with severe aplastic anemia, 4 with acute leukemia, 3 with acute lymphoblastic leukemia, and 4 with other diseases. All 21 recipients received antiviral prophylaxis. Among them, 20 underwent allogeneic HSCT (allo‐HSCT), and 1 underwent autologous HSCT (auto‐HSCT). The study was approved by the Ethics Committee of the Children's Hospital of Hebei Province.

Archived qPCR‐confirmed positive blood samples for CMV, EBV, HSV‐1/2, HHV‐6, hepatitis B, and hepatitis C [12, 13, 14] were obtained from Children's Hospital of Hebei Province and Hebei General Hospital.

Nucleic acids were extracted from 200 µL of whole blood using the QIAamp Blood DNA Mini Kit (Qiagen, Hilden, Germany) in accordance with the manufacturer's instructions. The extracted nucleic acids were eluted in 60 µL of nuclease‐free water and stored at −80°C until further use.

### 2.2 Primers and Probes Design for DO‐RAP Assay

The DO‐RAP assay simultaneously detected four viruses using two reaction tubes, each requiring a pair of RAA primers and a TaqMan probe per target. Assay 1 targeted CMV and EBV, while Assay 2 targeted HHV‐6 and HSV‐1/2. The DO‐RAP probe sequences were derived from previously published qPCR assays, targeting UL123 for CMV [12], BALF5 for EBV [13], U31 for HHV‐6 [13], and UL30 for HSV‐1/2 [12]. The RAA primers used in the DO‐RAP assay were designed specifically for this study. Hairpin formation, primer‐dimer potential, GC content, and melting temperatures were evaluated using Oligo7 and AmplifX software. Primer and probe specificity was further validated using BLAST (http://blast.ncbi.nlm.nih.gov/Blast.cgi). The primers and probes were evaluated for compatibility to ensure their suitability for use in duplex assays. All primers and probes were synthesized by General Biologicals (Anhui, China). The sequences of all primers and probes are listed in Table 1.

**Table 1:** Primers and probes used in this work.

Virus	Prime/Probe	Sequence (5′ to 3′)	Product size (bp)	Gene	Source
CMV	DO‐RAP‐F	CACCTTGTCTCTCTCYTCATCYARAATCTT		UL123	This study
	DO‐RAP‐R	TTTACCAAGAACTCAGCCTTCCCTAAGACC	150		This study
	qPCR‐F	CTGGGCATAAGCCATAATCTCATC			[12]
	qPCR‐R	CAGCCTTCCCTAAGACCACC	98		[12]
	Probe^a^	FAM‐TGCCGCCATGGCCTGACTGCAGCCA‐BHQ1			[12]
EBV	DO‐RAP‐F	CATGGCACCACATACCCCTGTTTATCCGAT		BALF5	This study
	DO‐RAP‐R	GAGGCAGACACMCACGGAAGCCCTCTRGAC	119		This study
	qPCR‐F	CGGAAGCCCTCTGGACTTC			[13]
	qPCR‐R	CCCTGTTTATCCGATGGAATG	90		[13]
	Probe^a^	CY5‐TGTACACGCACGAGAAATGCGCC‐BHQ3			[13]
HHV‐6	DO‐RAP‐F	CAATACTCTCTACTTATTCGACTCTCACCC		U31	This study
	DO‐RAP‐R	CGTCGTAGTAGAAGCCTTCRGTGCCGTGGG	131		This study
	qPCR‐F	TTTGCAGTCATCACGATCGG			[13]
	qPCR‐R	AGAGCGACAAATTGGAGGTTTC	223		[13]
	Probe^a^	FAM‐AGCCACAGCAGCCATCTACATCTGTCAA‐BHQ1			[13]
HSV‐1/2	DO‐RAP‐F	CACCGACCCGGAGAGGGACATCCAGGACTT		UL30	This study
	DO‐RAP‐R	CCGTCAGGTGGGCCAGGCGCTTGTTGGTGT	98		This study
	qPCR‐F	AGAGGGACATCCAGGACTTTG			[12]
	qPCR‐R	CTTGTAATACACCGTCAGGTGG	98		[12]
	Probe^a^	CY5‐ACCGCCGAACTGAGCAGACACCCGC‐BHQ3			[12]

^a^ Probe modifications: BHQ, black hole quencher; CMV, cytomegalovirus; CY5, cyanine 5; EBV, Epstein‐Barr virus; FAM, 6‐carboxyfluorescein; HHV‐6, human herpesvirus 6; HSV‐1/2, herpes simplex virus1/2.

### 2.3 Preparation of Recombinant Plasmids

A 300 bp fragment of the CMV UL123 gene (nt172981‐173280, GenBank accession no. NC_006273.2), a 300 bp fragment of EBV BALF5 (nt156181‐156480, GenBank accession no. OR652423.1), a 360 bp fragment of HHV‐6 U31 (nt46561‐46920, GenBank accession no. KY315550.2) and a 420 bp fragment of HSV‐1/2 UL30 (nt66209‐66628, GenBank accession no. NC_001798.2) were, respectively, cloned into the pUC57 vector to construct recombinant plasmids by TsingKe Biotech Corp (Beijing, China). Recombinant plasmids DNA was quantified using the Qubit dsDNA HS Assay Kits (Life technologies Invitrogen). Plasmid copy numbers were calculated using the following formula: Copy number (copies/µL) = {[6.02 × 10^23^ × plasmid concentration (ng/µL) × 10^−9^]}/[plasmid length × 660]. Serial 10‐fold dilutions of recombinant plasmids, ranging from 10^5^ to 10^0^ copies/µL, were used as standards for sensitivity analysis of the DO‐RAP assay.

### 2.4 Establishment of DO‐RAP Assay

The primary reagents used for the DO‐RAP assay consisted of RAA reagents and Taq DNA polymerase. The volumes of buffer, primer‐probe ratios, choice of qPCR enzyme, RAA reaction duration, qPCR annealing temperature, and magnesium ions concentration were systematically optimized and finalized.

**Figure 2:**
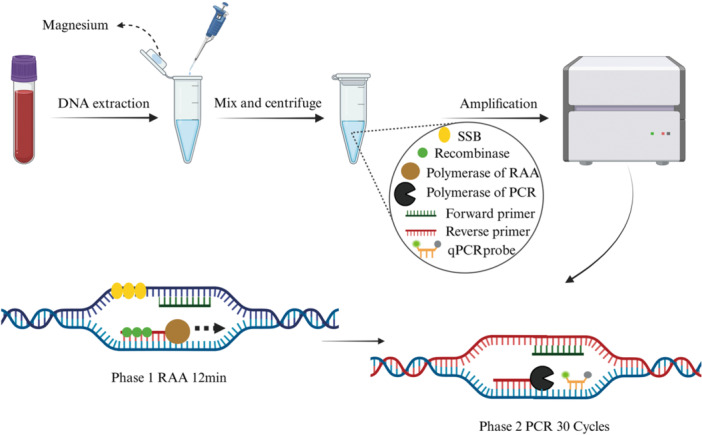
Schematic overview of the DO‐RAP assay. The assay includes an RAA amplification step followed by a PCR amplification step. This figure was created by BioRender.

The final 20 µL reaction mixture for Assay 1 included 8 µL of reaction buffer and RAA enzyme mixture (Amp‐Future, Amp‐Future Biotech Co. Ltd., Changzhou, Jiangsu, China), 1 µL (2.5 U) of Taq DNA polymerase (Nagene Diagnosis, Beijing, China), 0.4 µL each of CMV forward and reverse primers (10 µM), 0.2 µL of the CMV probe (10 µM), 0.8 µL each of EBV forward and reverse primers (10 µM), 0.4 µL of the EBV probe (10 µM), 0.8 µL of betaine (5 M; Sigma‐Aldrich, St. Louis, MO, USA), 3.2 µL of nuclease‐free water, 2 µL of 90 mM magnesium ions (supplied as 9 mM magnesium acetate), and 2 µL of recombinant plasmid or extracted genomic DNA. The 20 µL reaction mixture for Assay 2 contained 7 µL of reaction buffer and RAA enzyme mixture, (Amp‐Future, Amp‐Future Biotech Co. Ltd., Changzhou, Jiangsu, China), 1 µL (2.5 U) of Taq DNA polymerase, 0.2 µL each of HHV‐6 forward and reverse primers (10 µM), 0.2 µL of the HHV‐6 probe (10 µM), 0.4 µL each of HSV‐1/2 forward and reverse primers (10 µM), 0.2 µL of the HSV‐1/2 probe (10 µM), 0.8 µL of 5 M betaine, 5.6 µL of nuclease‐free water, 2 µL of 90 mM magnesium ions, and 2 µL of recombinant plasmids or extracted genomic DNA. Subsequent amplification was performed using a LightCycler 480 II (Roche). The thermocycling program was set as follows: 42°C for 12 min, 95°C for 2 min, followed by 30 cycles of 95°C for 5 s and 58°C for 50 s with real‐time fluorescence acquisition. The operational principle of the DO‐RAP assay is shown in Figure 2.

### 2.5 Sensitivity and Reproducibility of DO‐RAP Assay

Tenfold serial dilutions of mixed recombinant plasmids, ranging from 10^5^ to 10^0^ copies/µL, were prepared to assess the analytical sensitivity of the DO‐RAP assay in two independent reactions. Conventional qPCR assays (single‐plex qPCR) for CMV, EBV, HHV‐6, and HSV‐1/2 were performed in parallel using equivalent template amounts, according to previously published protocols [12, 13]. Reproducibility was evaluated by testing each plasmid dilution eight times, with nuclease‐free water serving as the negative control in each run.

### 2.6 Specificity of DO‐RAP Assay

Due to limited sample availability, the specificity of the DO‐RAP assay was evaluated using stored samples positive for CMV, EBV, HHV‐6, HSV‐1/2, hepatitis B, and hepatitis C, all of which had been previously confirmed by qPCR in our laboratory [12, 13, 14].

### 2.7 Detection of Clinical Samples

A total of 70 clinical whole blood samples were detected by DO‐RAP assay. Samples from HSCT recipients were collected at three time points: before transplantation (*n* = 21), 0–30 days post‐HSCT (*n* = 21), and 31–60 days post‐HSCT (*n* = 21). For comparison, qPCR assays described in previous studies [12, 13] were conducted in parallel using the same clinical extracts. To resolve discrepancies between the two methods, amplicons generated by conventional two‐step nested PCR for CMV, EBV, HHV‐6, and HSV‐1/2, as reported previously [15, 16], were subjected to sequencing.

### 2.8 Statistical Analysis

The 95% detection probability (limit of detection) for the DO‐RAP assay was calculated using probit analysis. Agreement between detection results were assessed using kappa statistics and the McNemar tests, with a *p*‐value < 0.05 considered statistically significant. All statistical analyses were performed using IBM SPSS Statistics version 21 (IBM Corp., Armonk, NY, USA).

## 3 Results

### 3.1 Sensitivity and Reproducibility of DO‐RAP Assay

**Figure 3:**
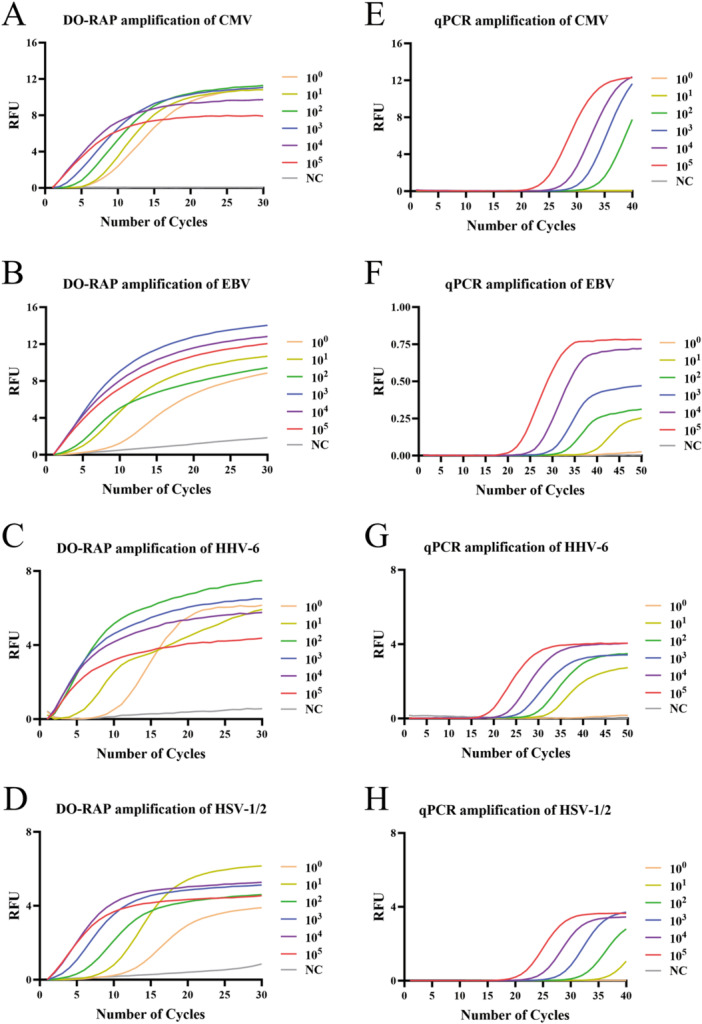
The sensitivity of DO‐RAP and qPCR were analyzed using recombinant plasmids of 10^0^–10^5^ copies/µL. The CMV (A), EBV (B), HHV‐6 (C), and HSV‐1/2 (D) recombinant plasmids were amplified by DO‐RAP; the CMV (E), EBV (F), HHV‐6 (G), and HSV‐1/2 (H) recombinant plasmids were amplified by qPCR. NC, negative control; cytomegalovirus (CMV), Epstein‐Barr virus (EBV), human herpesvirus 6 (HHV‐6), herpes simplex virus 1/2 (HSV‐1/2).

The analytical sensitivity was evaluated using 10‐fold serial dilutions of mixed recombinant plasmids ranging from 10^5^ to 10^0^ copies/µL. Each dilution was tested in eight independent replicates at various time points with the DO‐RAP assay, and the results are summarized in Supporting Information S1: Table S1. Probit analysis determined 95% detection limits of two copies/reaction for CMV, seven copies/reaction for EBV, two copies/reaction for HHV‐6, and three copies/reaction for HSV‐1/2 (Figure 3A–D). In comparison, the sensitivity of conventional qPCR ranged from 20 to 200 copies/reaction (Figure 3E–H).

### 3.2 Specificity of DO‐RAP Assay

To evaluate the specificity of the DO‐RAP assay, stocked positive samples for CMV, EBV, HHV‐6, HSV‐1/2, hepatitis B, and hepatitis C were tested. No cross‐reactivity with the DNA of any non‐target pathogen was observed (Supporting Information S1: Table S2). Additionally, no fluorescence amplification was observed in any negative controls, confirming the high specificity and analytical reliability of the DO‐RAP assay.

### 3.3 Evaluation Clinical Performance of DO‐RAP and qPCR Assay

The DO‐RAP assay was applied to a total of 70 clinical whole blood samples. Recipient samples were collected at three time points: before HSCT (*n* = 21), 0–30 days after HSCT (*n* = 21), and 31–60 days after HSCT (*n* = 21). For comparison, qPCR was performed simultaneously. Using DO‐RAP, 60 (85.71%) samples were positive for CMV, 42 (60.00%) for EBV, 12 (17.14%) for HHV‐6, and 54 (77.14%) for HSV‐1/2. In contrast, qPCR identified 47 (67.14%) CMV‐positive samples (Ct 30.43‐35.00), 28 (40.00%) EBV‐positive samples (Ct 25.92‐40.41), 8 (11.43%) HHV‐6‐positive samples (Ct 32.46‐35.81), and 48 (68.57%) HSV‐1/2‐positive samples (Ct 29.87‐35.00). A total of 37 samples were positive by DO‐RAP but negative by qPCR. Sequencing of amplicons generated by traditional two‐step nested PCR [15, 16] verified all 37 samples as true positives. As shown in Table 2, the DO‐RAP assay demonstrated 100% sensitivity for all four viruses, with specificities exceeding 43.48% relative to qPCR. Concordance between the two assays exceeded 80% for all viruses, and the kappa coefficients ranged from 0.51 to 0.79.

**Table 2:** Clinical performance of DO‐RAP in whole blood samples compared with qPCR results (*n* = 70).

DO‐RAP		qPCR		Sensitivity (%)	Specificity (%)	Agreement (%)	Kappa (*p* < 0.01)
		Positive	Negative				
CMV	Positive	47	13	100	43.48	81.43	0.51
	Negative	0	10				
EBV	Positive	28	14	100	66.67	80.00	0.62
	Negative	0	28				
HHV‐6	Positive	8	4	100	93.55	94.29	0.77
	Negative	0	58				
HSV‐1/2	Positive	48	6	100	72.73	91.43	0.79
	Negative	0	16				

**Figure 4:**
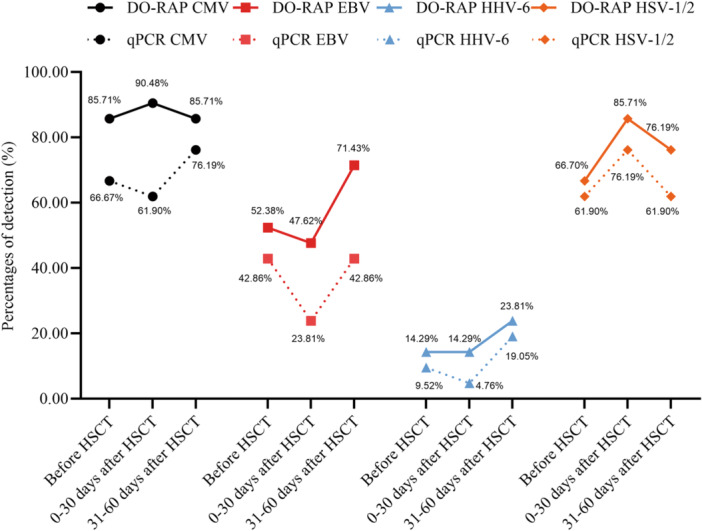
The percentages of detection of CMV, EBV, HHV‐6, and HSV‐1/2 by DO‐RAP and qPCR at different stages of HSCT recipients.

For whole blood samples collected from HSCT recipients before HSCT, the DO‐RAP assay demonstrated higher sensitivity than qPCR for CMV (85.71% vs. 66.67%), EBV (52.38% vs. 42.86%), HHV‐6 (14.29% vs. 9.52%), and HSV‐1/2 (66.70% vs. 61.90%). During the 0–30‐day period following HSCT, the DO‐RAP assay again showed higher sensitivity than qPCR for CMV (90.48% vs. 61.90%), EBV (47.62% vs. 23.81%), HHV‐6 (14.29% vs. 4.76%), and HSV‐1/2 (85.71% vs. 76.19%). Between 30 and 60 days post‐HSCT, the DO‐RAP assay maintained higher sensitivity than qPCR for CMV (85.71% vs. 76.19%), EBV (71.43% vs. 42.86%), HHV‐6 (23.81% vs. 19.05%), and HSV‐1/2 (76.19% vs. 61.90%) (Figure 4). The DO‐RAP assay successfully detected CMV, EBV, HHV‐6, and HSV‐1/2 in whole blood samples collected at three monitoring time points during HSCT. It demonstrated greater sensitivity than qPCR for monitoring the viral status of recipients before HSCT. The assay also proved superior sensitivity to qPCR for detecting viral reactivation across various post‐transplant stages. This study is a methodological validation study in which percentage differences were used to describe performance trends; statistical significance will be further verified in larger sample cohorts.

Among the seven donor samples, DO‐RAP identified one additional CMV‐positive donor (5/7, 71.43%) and one additional EBV‐positive donor (6/7, 85.71%) compared to qPCR. DO‐RAP detected HHV‐6 in one donor (1/7, 14.29%) and HSV‐1/2 in three donors (3/7, 42.86%), consistent with the qPCR results. As shown in Supporting Information S1: Table S3, DO‐RAP detected EBV in the donor from Case 1, whereas qPCR did not. These findings suggest that DO‐RAP exhibits greater sensitivity than qPCR for detecting CMV and EBV in donor whole blood. In the recipient from Case 1, DO‐RAP detected EBV and HSV‐1/2 earlier than qPCR. For the recipient in Case 2, DO‐RAP likewise enabled earlier detection of CMV, EBV, and HHV‐6 compared with qPCR (Supporting Information S1: Table S3). Overall, DO‐RAP demonstrates superior sensitivity to qPCR in recipient whole blood samples and enables earlier viral detection.

## 4 Discussion

Viral infections remain a major cause of morbidity and mortality in immunosuppressed patients following HSCT [17]. The seroprevalence of EBV in adults is approximately 95%. Primary EBV infection typically presents as infectious mononucleosis; however, after clinical recovery, the virus persists latently within B lymphocytes. Previous studies have shown that EBV DNA is consistently detectable in whole blood samples during symptomatic infection, but not uniformly detectable in plasma samples. Consequently, whole blood containing mononuclear cells is recommended for EBV DNA detection in transplant recipients [13, 17]. CMV infects 50%–80% of the population and establishes lifelong latency within monocytes. Whole blood and plasma specimens are equally suitable for CMV DNAemia monitoring; however, CMV DNA loads are usually higher in whole blood [17, 18]. HHV‐6 primarily infects CD4+ T lymphocytes and is also found latently in monocytes and macrophages. Detection of HHV‐6 DNA in whole blood may therefore reflect either latent or active infection [13]. The seroprevalence of HSV‐1 and HSV‐2 in adults is estimated at 50%–66.6% and 10%–20%, respectively [19, 20, 21]. In China, haploidentical transplantation (haplo‐HSCT) accounts for 60.1% of all HSCT cases, and post‐transplant viral infections are common [22]. Early and accurate monitoring of HSCT‐associated viral infections is essential for guiding clinical decision‐making and improving transplant outcomes. Here, a novel method was developed to enable rapid, accurate, and convenient detection of HSCT‐associated viral infections in whole blood samples.

The advantage of the DO‐RAP assay lies in its combination of the high sensitivity and rapid amplification of the RAA reaction with the straightforward probe design required for qPCR, thereby enabling a sensitive and rapid method capable of detecting multiple pathogens in a single tube. Previous studies have demonstrated that one‐tube RAP can be successfully applied to detect Candida and Mycobacterium tuberculosis infections in whole blood [23, 24, 25]. In this study, through optimization of reagent concentration and adjustment of reaction conditions, the DO‐RAP assay was adapted to simultaneously detect CMV, EBV, HHV‐6 and HSV‐1/2 in two tubes with high sensitivity, high specificity and reliable reproducibility (Figure 3, Supporting Information S1: Tables S1 and S2). Using 10‐fold serial dilutions of mixed recombinant plasmids, the assay achieved detection limits as low as 2 copies/reaction, representing a 10‐ to 100‐fold increase in sensitivity than qPCR (Figure 3).

Among the 70 clinical whole blood samples analyzed, DO‐RAP demonstrated 100% sensitivity and 43.48%–93.55% specificity relative to qPCR, with concordance rates exceeding 80% and Kappa values ranging from 0.51 to 0.79 (*p* < 0.01). The discrepancy observed between specificity and Kappa values is attributable to the exceptionally high sensitivity of the DO‐RAP method. A total of 37 samples tested positive by DO‐RAP but were negative by qPCR (Table 2). The qPCR Ct values ranged from 25.92 to 40.41, and more than half of these values were ≥ 35.00. In this study, recipients of HSCT received prophylactic acyclovir and letermovir immediately after transplantation to prevent viral infections. Consequently, virus loads in recipient whole blood samples were low, highlighting the superior sensitivity of the DO‐RAP detection over qPCR under low‐viral load situations. Furthermore, DO‐RAP can detect four viruses in two reactions within 62 min, at a per‐sample cost comparable to qPCR. Thus, this assay offers a rapid and highly sensitive approach for viral detection.

The DO‐RAP assay detected CMV, EBV, HHV‐6, and HSV‐1/2 in whole blood samples collected from recipients at three time points: before HSCT, 0–30 days after HSCT, and 31–60 days after HSCT. This assay demonstrated greater sensitivity than qPCR for detecting these viruses across all collection periods (Figure 4 and Supporting Information S1: Table S3). Previous studies indicate that CMV reactivation occurs in 18%–85% of patients, typically beginning around 2 weeks post‐HSCT, with a median onset of 32–41 days [26]. EBV reactivation occurs in 13%–82% of HSCT recipients, with a median time to first reactivation of 20 days (range: 3–244 days) after transplantation [27, 28]. HHV‐6 reactivation is observed in approximately 63% of patients by Day 100, with a median onset of 25 days after transplantation [29]. In this study, all four viruses were detectable at each time point, with the highest detection rates for CMV and HSV‐1/2 occurring at 0–30 days and for EBV and HHV‐6 at 31–60 days after HSCT (Figure 4). Monitoring these viruses in pediatric whole blood samples via DO‐RAP during HSCT enables earlier detection of viral reactivation, thereby facilitating timely clinical intervention.

In Case 1, DO‐RAP identified an EBV‐positive donor who tested negative by qPCR (Supporting Information S1: Table S3). During donor testing, DO‐RAP also detected one additional CMV‐positive case that qPCR failed to identify. Recipient monitoring in Cases 1 and 2 further demonstrated that DO‐RAP offers greater sensitivity and earlier detection of CMV, EBV, HHV‐6, and HSV‐1/2 than qPCR (Supporting Information S1: Table S3). According to the 2017 ECIL7 guidelines for the management of CMV infection in patients with haematological malignancies and post‐HSCT [18], monitoring with highly sensitive techniques like whole blood PCR allows for intervention before clinical CMV disease develops, and pre‐emptive therapy may be used alone or in combination with antiviral prophylaxis. Ganciclovir, valganciclovir, and foscarnet remain widely used for both CMV prophylaxis and pre‐emptive therapy in China; however, their use is often limited by significant toxicities [22]. The introduction of letermovir which has no major toxic effects has shifted CMV management in China from predominantly pre‐emptive therapy toward prophylaxis therapy. However, a 2023 study found that 17.6% of patients still experienced CMV reactivation after discontinuing letermovir [30]. Therefore, patients receiving letermovir prophylaxis require continued monitoring after drug discontinuation [18, 31]. For EBV, prophylaxis constitutes the use of acyclovir. Rituximab serves to treat reactivation effectively, lowering both the risk and mortality associated with PTLD [28]. Acyclovir prophylaxis also significantly reduces HSV infections in recipients of HSCT. However, acyclovir‐refractory or acyclovir‐resistant HSV infections can substantially worsen prognosis in allogeneic HSCT recipients [9]. For HHV‐6, surveillance followed by pre‐emptive treatment remains the primary management strategy, with ganciclovir and foscarnet serving as the first‐line antiviral options for HHV‐6 reactivation [29, 32]. In this study, DO‐RAP detected these viral infections in donors and recipients earlier than qPCR before HSCT, indicating its strong capability for early‐stage viral identification in the transplant setting. After HSCT, DO‐RAP continued to detect viral infections earlier than qPCR across all monitoring time points. This earlier detection facilitates timely preventive and pre‐emptive antiviral interventions, reduces donor‐recipient transmission risks, decreases the incidence of post‐transplant viral infections, and ultimately supports improved transplantation outcomes.

This study has several limitations. Firstly, the sample size was relatively small, likely due to the scarcity of donors, which in turn constrained both the number of transplantation cases and the number of specimens after transplantation. Secondly, DO‐RAP was unable to provide quantitative results, probably because the RAA amplification stage elevates template levels to the qPCR detection threshold. Future studies will aim to refine the RAP method to enable quantitative detection of pathogens. In addition, analyses of a larger number of samples from diverse specimen types and expansion of the pathogen panel are planned to provide a more comprehensive assessment and to further enhance the rapid and accurate detection of HSCT‐associated pathogens.

## 5 Conclusions

In conclusion, the DO‐RAP assay offers a sensitive, rapid, and operationally simple method for detecting major herpesviruses in pediatric HSCT recipients. Owing to its superior sensitivity compared with qPCR, DO‐RAP facilitates earlier viral identification, which is advantageous for both donor screening and post‐transplant surveillance. Earlier detection enabled by this assay supports timely clinical intervention and ultimately contributes to improved patient management.

## Author Contributions

**Li Zhao:** conceptualization, methodology, investigation, formal analysis, and writing – original draft. **Mengchuan Zhao:** methodology, investigation, and data curation. **Yuan Gao:** methodology, investigation, and formal analysis. **Yanqing Tie:** resources and validation. **Yinghui Guo:** formal analysis and validation. **Shuai Zhang:** data curation and validation. **Yuxin Wang:** investigation and formal analysis. **Deran Ma:** data curation and formal analysis. **Zhili Shao:** resources, conceptualization, methodology, investigation, and formal analysis. **Zhishan Feng:** resources, conceptualization, methodology, formal analysis, and writing – review and editing. All authors read and approved the final manuscript.

## Ethics Statement

All aspects of the study were performed in accordance with national ethics regulations and approved by the Ethics Committee of Children's hospital of Hebei Province (Lot Number: 2024169). The clinical samples used in this study were the remaining samples used for routine clinical testing and were completely anonymous, meeting the requirements of exemption from informed consent.

## Conflicts of Interest

The authors declare no conflicts of interest.

## Supporting information


**Supplementary Table S1:** The reproducibility of the DO‐RAP assay. **Supplementary Table S2:** The specificity of the DO‐RAP assay. **Supplementary Table S3:** Comparison of CMV, EBV, HHV‐6 and HSV‐1/2 detected by DO‐RAP and qPCR in two cases.

## Data Availability

All data are available in this manuscript. Additional information can be obtained from the corresponding author upon reasonable request.
