# Supplementary material for: Sensitive and Rapid Dual One‐Tube Recombinase Aided PCR Assays for the Simultaneous Detection of Cytomegalovirus, Epstein‐Barr Virus, Human Herpesvirus 6, and Herpes Simplex Virus 1/2 in Whole Blood From Children Undergoing Hematopoietic Stem Cell Transplantation
Source: J Med Virol. 2026 Apr 7;98(4):e70905. doi: 10.1002/jmv.70905 (PMC13054919; doi:10.1002/jmv.70905)
Supplement: Supplementary file 1 — Supplementary Table S1: The reproducibility of the DO‐RAP assay. Supplementary Table S2: The specificity of the DO‐RAP assay. Supplementary Table S3: Comparison of CMV, EBV, HHV‐6 and HSV‐1/2 detected by DO‐RAP and qPCR in two cases. [file JMV-98-e70905-s001.doc]

**Supplementary Table S1.** The reproducibility of the DO-RAP assay

| **copies/µl** | **CMV** | **EBV** | **HHV-6** | **HSV-1/2** |
| --- | --- | --- | --- | --- |
| 10^0^ | 8/8^a^ | 3/8 | 8/8 | 7/8 |
| 10^1^ | 8/8 | 8/8 | 8/8 | 8/8 |
| 10^2^ | 8/8 | 8/8 | 8/8 | 8/8 |
| 10^3^ | 8/8 | 8/8 | 8/8 | 8/8 |
| 10^4^ | 8/8 | 8/8 | 8/8 | 8/8 |
| 10^5^ | 8/8 | 8/8 | 8/8 | 8/8 |

^a^The number of positive results in 8 repeated experiments.

**Supplementary Table S2.** The specificity of the DO-RAP assay

| **Pathogen** | **CMV** | **EBV** | **HHV-6** | **HSV-1/2** |
| --- | --- | --- | --- | --- |
| cytomegalovirus | Positive | Negative | Negative | Negative |
| Epstein-Barr virus | Negative | Positive | Negative | Negative |
| human herpesvirus 6 | Negative | Negative | Positive | Negative |
| herpes simplex virus 1/2 | Negative | Negative | Negative | Positive |
| hepatitis B | Negative | Negative | Negative | Negative |
| hepatitis C | Negative | Negative | Negative | Negative |

**Supplementary Table S3.** Comparison of CMV, EBV, HHV-6 and HSV-1/2 detected by DO-RAP and qPCR in two cases

|  |  | **DO-RAP assay** | | | |  | **qPCR assay** | | | |
| --- | --- | --- | --- | --- | --- | --- | --- | --- | --- | --- |
|  |  | CMV | EBV | HHV-6 | HSV-1/2 |  | CMV | EBV | HHV-6 | HSV-1/2 |
| Case 1  donor | Before HSCT | negative | positive | negative | negative |  | negative | negative | negative | negative |
| Case 1  recipient | Before HSCT | positive | positive | negative | positive |  | positive | positive | negative | positive |
|  | 0-30 days after HSCT | positive | positive | negative | positive |  | positive | negative | negative | negative |
|  | 31-60 days after HSCT | positive | positive | positive | negative |  | positive | negative | positive | negative |
| Case2  recipient | Before HSCT | positive | negative | negative | positive |  | negative | negative | negative | positive |
|  | 0-30 days after HSCT | positive | negative | positive | positive |  | positive | negative | negative | positive |
|  | 31-60 days after HSCT | positive | positive | positive | positive |  | positive | negative | positive | positive |
